# Feasibility of magnetic resonance guided radiotherapy for the treatment of bladder cancer

**DOI:** 10.1016/j.ctro.2020.09.002

**Published:** 2020-09-11

**Authors:** A. Hunt, I. Hanson, A. Dunlop, H. Barnes, L. Bower, J. Chick, C. Cruickshank, E. Hall, T. Herbert, R. Lawes, D. McQuaid, H. McNair, A. Mitchell, J. Mohajer, T. Morgan, U. Oelfke, G. Smith, S. Nill, R. Huddart, S. Hafeez

**Affiliations:** a The Institute of Cancer Research, London, UK; b The Royal Marsden NHS Foundation Trust, London, UK; c The Joint Department of Physics at The Institute of Cancer Research and The Royal Marsden NHS Foundation Trust, London, UK; d Clinical Trials and Statistics Unit, The Institute of Cancer Research, London, UK

**Keywords:** Adaptive radiotherapy, bladder cancer, MR-guided radiotherapy, MR-linac

## Abstract

Whole bladder magnetic resonance image-guided radiotherapy using the 1.5 Telsa MR-linac is feasible. Full online adaptive planning workflow based on the anatomy seen at each fraction was performed. This was delivered within 45 min. Intra-fraction bladder filling did not compromise target coverage. Patients reported acceptable tolerance of treatment.

## Highlights

• Magnetic resonance image-guided whole bladder radiotherapy was feasible using a 1.5 T MR-linac.
• Each fraction was re-contoured, planned, and delivered in online workflow within 45 min.
• Intra-fraction bladder filling did not compromise target coverage.

## 1 Introduction

The commercial availability of combined magnetic resonance imaging (MRI) and radiotherapy units has fuelled pursuit of the clinical applications of magnetic resonance image guided radiotherapy (MRgRT) [1], [2], [3], [4], [5], [6]. MRI provides superior soft tissue contrast compared to standard onboard X-ray based imaging without the additional ionising radiation risk [7]. When combined with online adaptive replanning capabilities, MRgRT offers opportunity to adapt the plan at each fraction to the anatomical changes seen [8], [9].

The bladder is subject to large inter fractional position, shape, and size variation [10], [11], [12]. Historically large population-based planning target volume margins have been used in an attempt to achieve acceptable target coverage. This results in excessive normal tissue irradiation but does not successfully mitigate against geographical target misses [13], [14].

A number of adaptive radiotherapy solutions have been developed to address inter fractional bladder motion. The most widely reported approach is to generate a library of plans that model the expected spectrum of inter fractional bladder change. The plan with the best fitting dose distribution to the anatomy seen on cone beam CT (CBCT) acquired immediately prior to each fraction is then selected for treatment [14], [15], [16], [17]. Improved normal tissue sparing is seen with this technique compared to standard single plan treatment delivery [15], [18]. However, pre-clinical work demonstrates further dosimetric gains would be possible in bladder cancer radiotherapy with on-line replanning at each fraction [19], [20].

MRI has an established role in identifying muscle invasive bladder cancer as well as improving inter and intra observer delineation variation [7], [9], [21], [22], [23]. It is hypothesised therefore that MRgRT could address the systematic errors related to target definition by reducing the existing ambiguity of bladder tumour visualisation using CT and CBCT so facilitating future tumour boost and partial bladder irradiation approaches [20], [24], [25], [26].

The clinical feasibility of an MRgRT online re-optimisation approach using both the 1.5 Tesla (T) Elekta Unity (Elekta AB, Stockholm, Sweden) and the 0.35 T Viewray MRIdian (ViewRay Inc, Oakwood Village, OH) have been successfully demonstrated for a number of tumour sites [2], [8], [27]. A particular challenge for its clinical application in bladder cancer radiotherapy is the longer expected online adaptive workflow session times means that intra-fraction bladder filling has to be successfully accommodated for in order to maintain adequate target coverage.

We report the first clinical experience of MRgRT for the treatment of bladder cancer using the 1.5 T MR-linac. Our aim was to demonstrate initial clinical feasibility of full online planning based on anatomical change seen at each fraction, within a time frame of less than 60 min that was acceptable to muscle invasive bladder cancer (MIBC) patients unsuitable for radical treatment.

## 2 Methods and materials

### 2.1 Eligibility

Between April 2019 and December 2019, 5 patients with MIBC who were suitable for hypofractionated weekly radiation therapy but unsuitable for radical treatment with either cystectomy or daily radiotherapy due to either cancer stage or comorbidity were prospectively recruited to an institutional Clinical Research and Ethics Committee approved protocol for MRgRT (NCT03727698). All study participants gave written informed consent.

Patients with contra-indications to MRI, prosthetic hips or an inability to lie flat for the anticipated duration of an MRgRT treatment session were excluded.

### 2.2 Reference plan generation

Patients were asked to empty their bladder immediately prior to undergoing a non-contrast enhanced planning CT scan (CT_planning_) [15], [28], [29]. No drinking protocol was adopted. For those patients with a catheter in situ, the catheter was on free flow.

The clinical target volume (CTV) was contoured to encompass gross tumour volume (GTV), the whole bladder, and any extravesical spread. The CTV included 1.5 cm of the prostatic urethra (in males) or 1 cm of urethra (in females) if tumour was present at the bladder base or if distant CIS was present. In patients with direct invasion in to the prostate or co-existent prostate adenocarcinoma requiring treatment, the whole prostate was also included in the CTV. A planning target volume (PTV) was created by applying anisotropic margins to the CTV as follows: 1.5 cm anteriorly and superiorly, 1 cm posteriorly, and 0.5 cm laterally and inferiorly.

The organs at risk (OARs) were identified as the rectum, other bowel (includes both small and large bowel as single structure), and femoral heads. Details of target volume and OARs delineation used have been previously described [15], [28], [29].

Treatment planning system (TPS) Monaco (version 5.4, Elekta AB, Stockholm, Sweden) was used to create a 7-field, step and shoot intensity modulated radiotherapy (IMRT) reference plan on the CT_planning_. The reference plan included the effect of the 1.5 T magnetic field on dose distribution; it also functioned as an initial template for online re-planning, and provided relative electron densities for bulk density override regions of interest (ROIs) (Supplementray material Table 2) to facilitate MRI dose calculation.

Details of the bladder planning constraints and template parameters are provided in Supplementary Material; Table 1 and Table 2 respectively. The prescription dose (PTV D50%) was 36 Gy in six fractions delivered weekly; 30 Gy in five fractions was used for local symptom palliation in those with metastatic disease.

### 2.3 Online adaptive workflow

Patients were asked to void their bladder prior to set up. All patients underwent an online ‘Adapt-to-Shape’ (ATS) workflow with daily recontouring and plan reoptimisation [30], [31]. A transverse 3D T2-weighted (T2w) MRI with 2 min acquisition time (MRI_session_) was obtained, exported to the Monaco TPS, and registered to the CT_planning_ using soft tissue matching. Contours were propagated from CT_planning_ to MRI_session_ using rigid and deformable image registration (DIR). Rigid image registration was only used for a guide structure identifying the bladder base +/- prostate to ensure reproducibility of the inferior border of the CTV on each MRI_session_. The CTV was recontoured from scratch. The deformed rectum and bowel contours were reviewed and amended within 2 cm of PTV.

A new radiotherapy plan informed by the MRI_session_ contours was optimised using the reference plan parameters. The dose distribution and DVH was reviewed by the clinican. Target coverage compromise was not permitted at the expense of meeting OAR constraints (Table 1). Independent plan check was carried out by a physicist. Prior to beam on, a further 2 min T2w MRI (MRI_verification_) was acquired to confirm appropriate coverage of the target was maintained. If the CTV was not completely within the PTV on MRI_verification_ a subsequent ‘Adapt-to-Position’ (ATP) workflow was performed. Based on rigid registration between MRI_session_ and MRI_verification_, the segments from the ATS plan were shifted relative to the isocentre. The dose was then recalculated on the MRI_session_ optimising the weights of the segments based on the new position [31]. No actual couch shift occurs [32]. A final post treatment 2 min T2w MRI (MRI_post_) was acquired to enable offline assessment of intra-fractional CTV change and coverage.

**Table 1:** Patient characteristics.

	Patient 1	Patient 2	Patient 3	Patient 4	Patient 5
Age	86	88	86	73	88
Sex	Male	Male	Male	Female	Male
**Performance status (KPS)**	90	70	80	80	80
Cancer staging	• T3N0M0 bladder	• T4aN0M0 bladder	• T4bN1M0 bladder	• T2N0Mx bladder	• T2N2M0 bladder
			• T2N0M0 prostate	• T4N3M1	• T2N1M0 prostate
				• Ureter	
**Structures Included within the CTV**	• Whole bladder	• Whole bladder	• Whole bladder	• Whole bladder	• Whole bladder
	• Urethra (1.5 cm)	• Prostate	• Pelvic sidewall	• Urethra (1.0c m)	• Prostate
			• Prostate		
**Volume of CTV as on CTplanning (cc)**	106	134	175	87	334
Prescription dose	36Gy in 6f	36Gy in 6f	36Gy in 6f	30Gy in 5f	36Gy in 6f
Clinical Notes	–	Long term urinary catheter in situ	–	Metastatic upper tract cancer, bladder radiotherapy given for bleeding	–

KPS karnofsky peformance score.

### 2.4 Offline assessment

The CTV, rectum, and other bowel was re-countoured in their entirety on the MRI_verification_, and MRI_post_ images. For off-line assessment, this was performed by a single observer (AH) to eliminate inter clinician contouring variation [33]. CTV coverage was then recalculated on the MRI_verification_, and MRI_post_ anatomy. Acceptable CTV coverage was as defined 95% of CTV receiving ≥95% of prescribed dose.

Conformity index (CI_RTOG_) was used as a surrogate measure of normal tissue irradiation [26], [34]. It was defined here as the proportion of the total volume receiving 95% dose compared to the volume of CTV. (i.e. CI_RTOG_ = volume of 95% isodose / volume of CTV). The higher the CI_RTOG_ value, the greater the proportion of normal tissue receiving >95% of the prescribed dose. Where CI_RTOG_ = 1, no normal tissue received >95% of the prescribed dose.

### 2.5 Patient experience

Patients completed a patient experience questionnaire following fractions 1, 2, 3, and on treatment completion. This tool was adapted by HM from both The Radiotherapy Experience Questionnaire and Magnetic Resonance Imaging-Anxiety Questionnaire [35], [36]. It consisted of items reflecting the patient’s comfort, coping, and informational needs during their MR-linac treatment and was scored on a 4-part Likert scale. A copy of the finalised tool used is available in the Supplementary material.

## 3 Results

Patient characteristics are summarised in Table 1. The median age was 86 years (range 73–88). All patients had transitional cell carcinoma of the bladder, 2 patients also had adenocarcinoma of the prostate, and one patient had metastatic disease. One patient was treated with a urinary catheter in situ (Patient 2).

### 3.1 Online adaptive workflow

All patients completed their planned course of treatment on the MR-linac. All 29 fractions were delivered using ATS protocol. Four fractions required additional ATP after ATS.

**Fig. 1:**
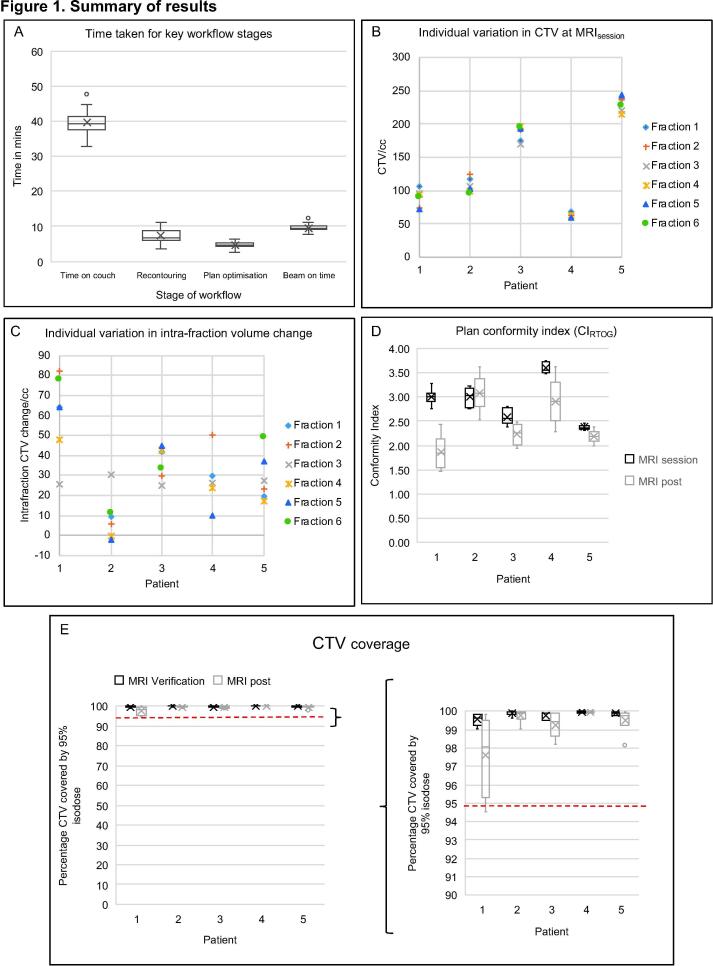
Summary of results. Panel A: Time taken for key workflow stages. Box and whisker plot where x is mean value, horizontal line is median value, box edges are interquartile range, whisker edges are the maximum and minimum values, and circles are outliers. Panel B: Inter- and intra-patient variation in CTV as seen on MRI_session_ image. Panel C: Intra-fraction CTV change, note for patient 2 volume decreases occurred at some fractions likely due to presence of urinary catheter (on free flow). Panel D: Plan conformity index (CI_RTOG_) comparing CI_RTOG_ of plan at time of MRI_session_ image compared to estimated delivered dose on MRI_post_ imaging. Aiming for a value approaching 1. Panel E: CTV coverage based on estimated dose delivered to the anatomy as seen at MRI_verification_ and MRI_post_ time points, magnified graph on the right. Aiming for CTV coverage to be >95% (dotted line), achieved in all fractions apart from Patient 1 fraction 6.

Fig. 1 summarises the time taken for key parts of the treatment workflow. All fractions were delivered in less than 60 min; median time on treatment couch was 39 min (range 33–48), during which the median recontouring time was 7 min (range 4–11), median plan reoptimisation was 5 min (range 3–6) and median treatment delivery 9 min (range 8–12).

For Patient 1, technical issues resulted in premature beam termination during fraction 4. The undelivered dose (15% of that fraction’s planned dose) was compensated for in the remaining fractions with a prescription dose increase. Patient 5 experienced a one week delay prior to fraction 3 due to a non-treatment related hospital admission. Compensation for this missed treatment was made by extending total treatment time by one week.

In Patients 2 and 4, the propagated external ROI from the CT _planning_ scan did not match the external contour of the MRI_session._ In order to correct this, a new reference plan for these patient was created using the fraction 1 MRI_verification_ image. When using the MRI based reference plan, propagation of the external ROI between the reference plan and the MRI_session_ was improved.

### 3.2 Inter- and intra-fraction target variation

The median CTV as determined on MRI_session_ was 107 cc (range 60–243 cc). Median intra-fraction CTV change (a surrogate for bladder filling as determined by change in volume between MRI_session_ and MRI_post_) was 30 cc (range −2–82 cc). Inter- and intra-fraction CTV variation is shown in Fig. 1.

### 3.3 Target coverage

CTV coverage was achieved for 28/29 fractions at verification and post treatment assessment. For 1/29 fractions the post treatment coverage was 94.5% (Patient 1) (Fig. 1). In 27/29 fractions, the estimated dose to OARs of the delivered plan was within the mandatory dose constraints based on the MRI_verification_, and MRI_post_ anatomy.

The OAR constraint violations occurred for Patient 2 fraction 1, when the V36Gy for other bowel exceeded tolerance on the MRI_verification_ (by 15 cc) but was within tolerance on MRI_post_ and subsequent fractions; and for Patient 5 fraction 2, when intra-fraction rectal distention resulted in rectal dose constraints to be exceeded by up to 12%, but remained within tolerance for all subsequent fractions.

The mean CI_RTOG_ on MRI_session_ was 2.9 (SD 0.44, range 2.3–3.8), and on the MRI_post_ mean CI_RTOG_ was 2.4 (SD 0.56, range 1.5–3.6) (Fig. 1).

### 3.4 Patient tolerability

The MR-linac questionnaire was completed by all patients at ≥ 3 time points. A total of 19/20 (95%) questionnaires were returned. Of the 235 items answered, 233 (99.1%) responses reflected acceptable/favourable treatment experience.

## 4 Discussion

We successfully demonstrate that full online adaptation for the treatment of bladder cancer on the 1.5 T Elekta Unity is technically and clinically feasible.

The time taken to deliver this workflow is comparable to the reported workflow treatment times for other tumour types on this platform [2]. It was well tolerated by patients with no sessions requiring termination due to distress or discomfort. This was particularly important as eligible patients were unsuitable for radical treatment either due to frailty, co-morbidity, or disease stage. This patient population was selected for feasibility testing as the weekly fractionation schedule enabled any inter-fraction technical issues to be resolved without adverse impact to the radiotherapy schedule.

The time taken for the workflow was an important consideration when determining the most appropriate margins to create the PTV in order that it would successfully accommodate for intra-fraction bladder filling. The margin used was derived from analysis (unpublished) of similar patient cohort treated in non-randomised phase I/II study (NCT01000129; ISRCTN80815524) in whom bladder filling had been observed over 30 min [15], [28], The anisotropic margins used here encompassed >90% of the intra-fraction bladder change over 30 min. A smaller, 0.5 cm isotropic margin encompassed only 68% of intra-fractional excursions over 30 min.

All patients demonstrated intra-fraction CTV change including Patient 2 who had a urinary catheter in situ. The magnitude of intra-fraction volume change varied both on an inter-patient and inter-fraction basis. No clear pattern of bladder filling was identified. The margins used successfully maintained target coverage in all but one fraction. This fraction showed the second highest intra-fraction volume change.

The mean CI_RTOG_ was used as a surrogate measure to illustrate normal tissue irradiation. For bladder radiotherapy delivered using CBCT ‘plan of the day/library of plans’ approach the similarly derived mean CI_RTOG_ was 3.5 [26]. This suggests that even without any margin reduction, adaption based on inter-fraction target varation alone offers potential dosimetric gains. In our current study the mean CI_RTOG_ as assessed on the MRI_post_ anatomy is more favourable (mean CI_RTOG_ 2.4). Trend in improved mean CI_RTOG_ was seen between corresponding MRI_session_ and MRI_post_ scans. Greatest improvement occurred when intra-fraction bladder filling occurred. It was not evident for Patient 2 with urinary catheter.

Given length of the current workflow, global margin reduction to 0.5 cm is unlikely to sufficiently maintain intra-fractional target coverage, therefore future work will investigate predictors of individual patient bladder filling in order to personalise the intra-fraction margin to further improve normal tissue sparing whilst maintaining target coverage.

The study has now been extended to include radical patients receiving daily whole bladder radiotherapy (55 Gy in 20 fractions). The possibility of using MRgRT to target the bladder tumour only is attractive as it opens opportunity to reduce toxicity and facilitate dose escalation. Tumour focused partial bladder radiotherapy can be utilised with no adverse effect on local control but it has failed to show clinical improvement in normal tissue toxicity when randomised against whole bladder treatment [37], [38]. One likely contributing factor is that the expansion margin of 1.5 cm applied around the GTV to create the PTV boost leaves very little normal tissue sparing compared to whole bladder treatment. MRgRT may lead to reduction in toxicity given modelling work to date demonstrates that MRI defined bladder tumour is up to 50% smaller than that defined on CT [39]. This translates to significant improved normal tissue sparing (>60%) at high bowel and normal bladder constraints compared to CT based delineation when escalating bladder boost dose to 70 Gy [40].

Daily replanning using the workflow described has required the presence of one physicist, two radiographers, and a clinician to deliver each fraction. Streaming this workflow with particular focus on radiographer led contouring is part of ongoing work [41].

## 5 Conclusion

We have successfully demonstrated that whole bladder magnetic resonance image-guided radiotherapy using the 1.5 T MR-linac is feasible. Full online adaptive planning workflow was delivered within 45 min. Intra-fraction bladder filling did not compromise target coverage. Patients reported acceptable tolerance of treatment.

## 6 Contribution

All authors meet at least of one the criteria recommended by the ICMJE. AH and SH wrote the first draft of the manuscript. All authors were involved in protocol development and contributed to subsequent revisions of the manuscript.
