# Supplementary material for: Feasibility of magnetic resonance guided radiotherapy for the treatment of bladder cancer
Source: Clin Transl Radiat Oncol. 2020 Sep 11;25:46–51. doi: 10.1016/j.ctro.2020.09.002 (PMC7522378; doi:10.1016/j.ctro.2020.09.002)
Supplement: Supplementary data 1 [file mmc1.pdf]

Supplementary material
Table 1. Dose volume constraints

Target volume constraints

Structure	Constraint to be achieved	
**PTV**	D95%	>95% prescribed dose
	D98%	>95% prescribed dose
	D99%	>90% prescribed dose

i.e where PTV D95% is the dose received by 95% of the PTV

Organ at risk constraint guide

Structure	Constraint to be achieved*		
	Dose level	Optimal	Mandatory
**Rectum**	V17Gy	<50%	<80%
	V28Gy	<20%	<60%
	V33Gy	<15%	<50%
	V36Gy	<5%	<30%
**Other Bowel**	V25	<139cc	<208cc
	V28	<122cc	<183cc
	V31	<105cc	<157cc
	V33	<84cc	<126cc
	V36	<26cc	<39cc
**Femoral head (left)**	V28Gy	-	<50%
**Femoral head (right)**	V28Gy	-	<50%
**Normal tissue (External -PTV)**	D1cc	<105% prescribed dose	<110% prescribed dose

*OARs constraints were as used in the preceeding phase I/II studies (NCT01000129 and NCT01810757) [1-3].

Supplementary material

Table 2: Radiotherapy plan template settings

Setting	Value
Number of beams	7
Dose scoring	Dose to medium
Grid spacing	0.3cm
Statistical uncertainty	2% per plan
Fluence smoothing	Medium
Maximum segments	80
Minimum segment width	0.5cm
Minimum segment area	$4cm^2$
Minimum segment monitor unit	3MU
IMRT constraints	PTV, Rectum, RectumSpare, Other_Bowel, BowelSpare, External_reduced
Bulk density ROIs	Bones_reduced (density taken from the bone ROI limited to superior and inferior extent of target), CTV, External_reduced (density taken from the External_reduced ROI limited to superior and inferior extent of target)
SSO loops	5

Where RectumSpare = Rectum – (PTV +1.5cm), planning structure to aid optimisation; BowelSpare = Other Bowel – (PTV+1.5cm), planning structure to aid optimisation**;** External_reduced = reduced volume external ROI to facilitate reduced treatment planning time; Bones_reduced = reduced volume bones ROI to facilitate reduced treatment planning times; ROIs= regions of interest; SSO = segment shape optimization.

Supplementary material

MR-linac patient questionnaire (version 0.3 dated 22.01.19)

Study Name:____________ Study Number: Date:_______________

Patient Experience Questionnaire; MRLinac (Unity) Radiotherapy treatment

Radiotherapy treatment delivered on the MR Linac (Unity) using Magnetic Resonance Imaging (MRI) is a new technology. We would like to find out your views about having treatment on Unity. This may help us improve the experience for you and other patients.

We would be grateful if you would complete this questionnaire after your treatment and return it to us before you leave.

Please circle the response that best fits your experience

	0 Not at all	1 Slightly	2 Moderately	3 Very
I needed more detailed information before my treatment	Not at all	Slightly	Moderately	Very
I found the treatment position comfortable	Not at all	Slightly	Moderately	Very
I found the treatment bed comfortable	Not at all	Slightly	Moderately	Very
I found it easy it to stay still and maintain the treatment position	Not at all	Slightly	Moderately	Very
I wanted to come out of the machine during my treatment	Not at all	Slightly	Moderately	Very
I felt calm during my treatment	Not at all	Slightly	Moderately	Very
I found the noise in the room easy to tolerate	Not at all	Slightly	Moderately	Very
I found the lighting in the room easy to tolerate	Not at all	Slightly	Moderately	Very
I found the time taken for the treatment easy to tolerate	Not at all	Slightly	Moderately	Very
I felt dizzy during my treatment	Not at all	Slightly	Moderately	Very
I felt dizzy immediately after my treatment	Not at all	Slightly	Moderately	Very
I felt hot during my treatment	Not at all	Slightly	Moderately	Very
I felt tingling sensations during my treatment	Not at all	Slightly	Moderately	Very
I experienced a metallic taste during my treatment	Not at all	Slightly	Moderately	Very
I needed more communication from staff during my treatment	Not at all	Slightly	Moderately	Very
I forced myself to manage the situation	Not at all	Slightly	Moderately	Very
I found listening to the music helpful whilst having my treatment	Not at all	Slightly	Moderately	Very
I understood the procedure	Not at all	Slightly	Moderately	Very
**Comments:**				

The MR-linac patient questionnaire was adapted from both The Radiotherapy Experience Questionnaire and Magnetic Resonance Imaging-Anxiety Questionnaire [4, 5] by Dr Helen McNair.
